# Alcohol management plans in Indigenous communities in Queensland (Australia) may have unintended implications for the care of children

**DOI:** 10.1186/s40352-016-0039-5

**Published:** 2016-07-18

**Authors:** Katrina Bird, Michelle S. Fitts, Alan R. Clough

**Affiliations:** Community-based Health Promotion and Prevention Studies Group Australian Institute of Tropical Health and Medicine James Cook University, Cairns, 4870 QLD Australia

## Abstract

**Background:**

Indigenous children in Australia are more likely than non-Indigenous children to be in contact with the child safety system. A large number of Queensland’s Indigenous population live in remote and isolated communities in north Queensland where the state government's Alcohol Management Plans (AMPs) are in effect. In these communities it is an offence to have in one’s possession more than the regulated amount and type of alcohol. A breach of these restrictions can result in convictions under the *Liquor Act 1992*.

**Findings:**

During an evaluation of AMPs, influential stakeholders and key service providers voiced their belief that a conviction for a breach of the AMP would impact a person’s eligibility to hold a Positive Notice Blue Card (PNBC). On its own, however, a breach of the *Liquor Act 1992* will not impact a person’s eligibility for a PNBC. A PNBC is required for any person volunteering or working with children. Without a PNBC, a person is ineligible to work in child-related employment, volunteer at child-related activities or provide out-of-home care for children.

**Conclusion:**

This misconception needs to be addressed in these already-disadvantaged communities to ensure that Indigenous community members have every opportunity to hold a PNBC. Focused strategies with evaluation and research are needed in this important policy area.

## Introduction

In the Australian state of Queensland, Aboriginal and Torres Strait Islander (Indigenous) children are over-represented in the child safety system, and were 8.3 times more likely than non-Indigenous children to be in out-of-home care in 2013/14 (Australian Institute of Health and Welfare, 2015). In Queensland, a Positive Notice Blue Card (PNBC) is a requirement for all persons working in *any* child-related employment, whether on a paid or volunteer basis, as well as for *all* foster and kinship carers (Commission for Children and Young People and Child Guardian 2014a; State of Queensland Public Safety Business Agency 2014; Tilbury, 2014). At the 2011 census, there were 69,156 Indigenous children in Queensland, of these, 9.5 % (6,537) lived in the 19 remote and isolated, discrete communities where the Queensland Government implemented alcohol control policies from 2002 onwards (Australian Bureau of Statistics, 2011).

These alcohol control policies are known as Alcohol Management Plans (AMPs). AMPs are regulated by the state's *Liquor Act 1992 (*the *“Liquor Act”*) and feature controls on the quantity and type of alcohol that can be possessed in these communities, making it an offence to have in one’s possession more than the prescribed amount or type of liquor (Clough & Bird, 2015; "The *Liquor Act* 1992,"; "The *Liquor Regulation* 2002 *(Qld)*,"). Persons convicted of *Liquor Act* offences can receive a criminal conviction (Queensland Government, 2010).

In the early stages of a formal evaluation of these alcohol control policies (Clough et al., 2014) it was found that key stakeholders, service providers and residents in some communities held a view that having a conviction for a breach of alcohol restrictions would limit a person’s eligibility to hold a PNBC. There was a parallel view expressed that a person’s capacity to work in child-related employment or activities, or to provide foster care to Indigenous children in need, would be limited. This matter does not appear to be clearly understood in these settings as a breach of the *Liquor Act,* in and of itself, does not in fact, constitute a disqualifying offence (*Working with Children (Risk Management and Screening) Act* 2000).

Any confusion about this extremely important matter could negatively impact the effectiveness of the child safety system for this group of already-disadvantaged Indigenous Australian children (Commission for Children Young People and Child Guardian, 2014). Because of the serious implications for the care and wellbeing of children, this paper attempts to describe and clarify the interactions between the formal requirements for a PNBC and the status of convictions under the *Liquor Act* for breaches of liquor restrictions in Queensland’s AMP communities. We first describe the PNBC application process, highlighting the natural justice principles followed in the assessment of a person’s criminal history. We then summarise the evidence compiled from interviews with stakeholders and service providers and surveys with community residents to provide a preliminary understanding of how the confusion may have come about, thereby providing opportunities to consider options for systematically documenting the extent of any confusion and opportunities to address it.

### Populations and living circumstances

The 2011 census data indicates that the 19 Indigenous communities in which AMPs were implemented have very small populations (17,485 people) (Australian Bureau of Statistics, 2011). Although there are some non-Indigenous people living and working in these communities, most residents are Indigenous (92.4 % = 16,163/17,485). In 2011, the total Indigenous population of these communities was made up of 6,537 (40.4 %) children under 18 years of age, and 9,626 (59.6 %) adults (Australian Bureau of Statistics, 2011). The Indigenous residents in these communities were accommodated in 3,135 dwellings (Australian Bureau of Statistics, 2011). With dwellings in these communities housing, on average, 3.1 adults (=9,636/3,135) and 2.1 children (=6,537/3,135), or 5.2 persons per dwelling overall. This reflects crowded living circumstances in comparison to Queensland’s general population where the average is around half this (2.8 persons per dwelling) (Australian Bureau of Statistics, 2011). One quarter of the dwellings in AMP communities house multiple families compared with just 2.1 % for Queensland as a whole (Australian Bureau of Statistics, 2011). More than two-thirds (69 %) of dwellings house four or more residents, double the rest of Queensland (34 %) (Australian Bureau of Statistics, 2011).

### Liquor regulation in restricted areas – indigenous communities with AMPs

In the late 1990s and early 2000s, several reviews of the specific impacts of alcohol in these Indigenous communities revealed that alcohol availability and consumption had compromised the safety, health and welfare of community members (Fitzgerald, 2001; Martin, 1998; Martin & Brady, 2004; Queensland Government, 2002). Residents of these communities were seen by policy makers to be living *“a lifestyle dominated by alcoholism and substance abuse”* (Queensland Government, 2002 [p. 6]) compared with other parts of Queensland (Green, 2001; Queensland Government, 2002) with alcohol, substance abuse and violence responsible for general social dysfunction in these communities (Fitzgerald, 2001). In 2001, the *Cape York Justice Study* [CYJS] concluded that children in these Indigenous communities were experiencing violence-related trauma, neglect, physical and sexual abuse along with poor health (Fitzgerald, 2001 [CYJS Vol 1 p44]). To address what was described as “*sickness, suffering and fear*”, [MCMC, *p9*] with children as a focus, the Queensland Government introduced AMPs at different stages between December 2002 and June 2006 across 19 targeted communities (Clough & Bird, 2015; Queensland Government, 2002).

These communities are declared “restricted areas” under part 6A of the *Liquor Act.* The type and quantity of alcohol that can be possessed in these restricted areas are outlined in Schedules to The *Liquor Regulations* 2002 (Clough & Bird, 2015; Liquor Regulation 2002 (Qld)). It is an offence to have in one’s possession more than the prescribed amount or type of liquor in these communities, with the possibility of a criminal conviction being recorded (Clough & Bird, 2015; Queensland Government, 2012; The *Liquor Act* 1992).

### Method and approach

Information from this paper has been drawn from a large evaluation study aiming to describe the impacts on important health, economic and social outcomes of the AMPs (Clough et al. 2014). Key stakeholders and service providers in the region were interviewed (*n* = 382) if they had a current or past role in a service with either direct or indirect responsibility for managing the issues and consequences surrounding AMPs. All those interviewed were asked open-ended questions seeking their views on favourable or unfavourable impacts of AMPs that they had witnessed or experienced. Complementing these interviews (Clough et al. [in press]), community residents in 10 communities where AMPs operate completed a survey (*n* = 1211). Clough et al., 2014 provides a more detailed description of the study.

### The Blue Card application process in Queensland

To provide an efficient summary of the operations of the Blue Card system, the authors reviewed available on-line documents, relevant publications and policy statements and consulted with Blue Card Services, Public Safety Business Agency, Queensland.

## Child safety

### General principles and guidelines

In Australia if a child becomes at risk of significant harm, child protection authorities are obliged to intervene (Australian Institute of Family Studies, 2015), with out-of-home care provided when children are unable to live with their parents (Australian Institute of Health and Welfare, 2014; 2015). When determining the type of out-of-home care for Indigenous children, Queensland’s Department of Child Safety must give consideration to a culturally appropriate placement in accordance with the Indigenous Child Placement Principal (ICPP) (Child Protection Act, 1999: S83). The ICPP supports Indigenous families and communities to influence decisions about their children (Commonwealth of Australia et al., 2015). Kinship care, where the child is cared for a by a relative or close member of the community, is the preferred placement option for Indigenous children (Blacklock et al., 2013) with priority of placement to Indigenous carers to ensure children are cared for within their community *“in a way that respects their culture and helps them maintain their cultural identity”* (Queensland Government, 2013).

### The blue card system: working with children checks in Queensland

**Fig. 1:**
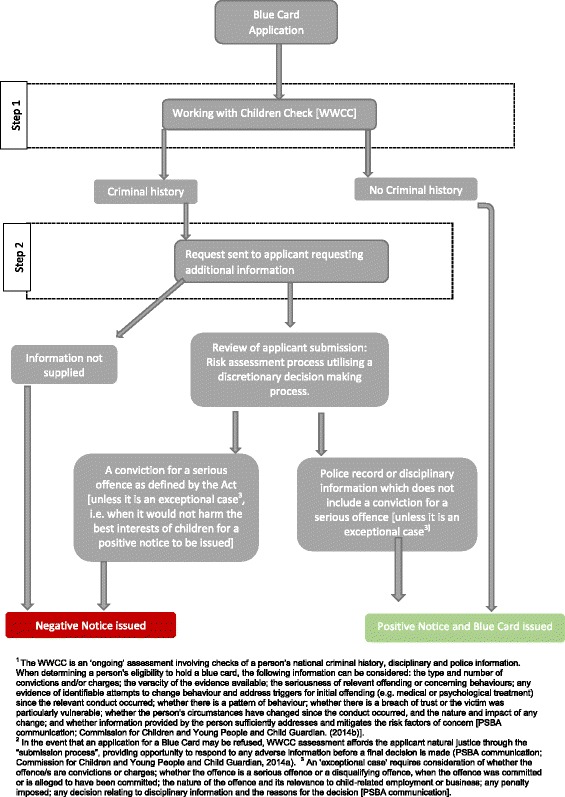
Positive Notice Blue Card application process

A PNBC is a requirement for all persons working in child related employment on a paid or volunteer basis as well as all foster and kinship carers in Queensland, a requirement which is applied to all adult members who reside in the proposed foster/kinship household, along with regular visitors (Commission for Children and Young People and Child Guardian 2014a; State of Queensland (Public Safety Business Agency [PSBA]), (2014)). A person applies for a PNBC through Blue Card Services, PSBA. The application process (see Fig. 1) includes a Working With Children Check (WWCC) conducted under the *Working with Children (Risk Management and Screening) Act* 2000.

The WWCC is an ‘ongoing’ assessment of a person’s eligibility to work or volunteer with children (Commission for Children and Young People and Child Guardian 2014; State of Queensland (PSBA), (2014)) and involves a check of a person’s national criminal history, disciplinary and police information (Commission for Children and Young People and Child Guardian 2014a; State of Queensland (PSBA), (2014); Tilbury, 2014). Convictions and/or charges considered include, but are not limited to, sexual offences against children, murder, manslaughter, domestic violence, assault including sexual assault, drink driving and behaviours related to it, and drug offences (Tilbury, 2014; *The Working with Children (Risk Management and Screening) Act* 2000).

When engaging Indigenous people in the Blue Card system the PSBA have identified several challenges, including remoteness/isolation, establishing contact, language barriers, literacy, cultural issues and proof of identity (Commission for Children Young People and Child Guardian, 2014), all of which present significant challenges when dealing with the “submission process” (Fig. 1). As a result, many applicants in Indigenous communities seek the assistance of key stakeholders and service providers within their communities to assist in the application/submission process.

## Findings

### Views of key community service providers and stakeholders

During interviews, key stakeholders, service providers and influential community leaders working in the areas of justice and Indigenous children's advocacy frequently asserted the view that criminal convictions resulting from *Liquor Act* breaches make it difficult for community members to achieve a PNBC. Although the AMP evaluation study did not specifically seek to investigate this complex matter (Clough et al., 2014), the quotes below illustrate this view was held across various workforce sectors:

*“… I am concerned about the criminality of alcohol… it’s one thing to go through the courts and get a fine, but as soon as you start getting convictions recorded… the inability to get blue cards, the way Indigenous communities work in placing kids around family to make sure they get the best upbringing they can… like blue cards, you can’t get that.” (Education)*

*”it’s going to be hard for them to get a job, you can’t get a passport for anything like that yeah… it is going to be hard for them, I mean it’s hard to get a job now because you have got to get a blue card to work with children so it will be even harder if they get a criminal record.” (Government)*

Moreover, these views were shared by a small number of survey participants in qualitative comments recorded verbatim on their survey forms, for example:

*“It’s not helping them, how will it affect them when they go for a job, blue cards.” (Community resident)*

For communities there are further implications to consider including the long-term impacts of increases in the criminal records held by adults, effects on employment and the challenge of addressing foster caring in impoverished and overcrowded living circumstances.

### Criminal records

According to publically available Queensland Government data, since the implementation of AMPs and up to the 30^th^ of June 2014, 6,961 people had been convicted of 15,511 charges for breaches of S168B and/or S 168C of the *Liquor Act* (Queensland Government, 2014). Persons attempting to bring alcohol into a restricted area are charged under a breach of S168C, whereas possession of prohibited quantities or types of alcohol in a restricted area are charges under S168B. Almost all the people charged were Indigenous residents of AMP communities meaning that approximately 70 % of the adult resident Indigenous population of the affected 19 communities had accrued, by this time, at least one charge of breaching liquor restrictions. This is reflected in the following impactful statement:

*“… you can't get a blue card… you got a criminal history, what poor soul in this community hasn't got a criminal history.” (Justice)*

### Employment

With a large proportion of the limited available job opportunities in the community involving contact with children including: employment at the community school; sports and recreation functions; child care and health services, the perception of being ineligible to attain a blue card potentially adds to the significant challenges adults face when seeking work:

*“And that stays with you, and there’s nothing you can do, you can’t get jobs, you can’t get jobs in child safety because of that, so it really restricts your job opportunities.” (Justice)*

*“So we have got people who have got no criminal history, all of a sudden have a criminal history* [due to the AMP]*… When we say to these women, do you know, you can’t go and work at the day care centre or the school anymore because now you have a criminal history and you can’t get a blue card.” (Health)*

### Care of children

As at 30 June 2014, for every 1,000 Indigenous children in Queensland, 25.5 were subject to child protection substantiations compared to non-Indigenous children whose rate was 4.1 per 1,000 (Australian Institute of Family Studies, 2015). Indigenous children are 8.1 times more likely than non-Indigenous to be subject to a care and protection order (42.2 per 1,000 compared to 5.2 per 1,000) (Australian Institute of Health and Welfare, 2014). In the Indigenous communities subject to an AMP (for the period 1 July 2013 to 30 June, 2014) the estimated average rate for children (aged 0–17) subject to a substantiation order was 28 per 1,000 (Queensland Government, 2014). These high rates demonstrate the significant demand for suitable foster carers, further highlighting the need to eliminate any perceived barriers to finding them:

*“We are flat out getting foster carers approved because of the AMP.” (Welfare worker)*

*“AMPs have created a long court list, criminals. Later on these guys might become great men and women, and can't become carers.” (Welfare worker)*

### Overcrowding

A child will not be placed with a carer if any member of their household is ineligible to hold a PNBC (Moynihan et al., 2006). With the number of adults and children living in a household at around twice the state average, extreme measures, potentially in breach of regulations, are sometimes used to overcome the requirement for every adult to hold a PNBC:

*“You see one of the things that happens they will place a child with a person that doesn't have approval under a safety plan. But as soon as the Department has an order for that child it is a legislative requirement that that person and all members of their household have blue cards. We're getting fairly adept at lying about who are members of the household. [….] That's a very high barrier, cs.” (Justice)*

#### Indigenous engagement with the Blue Card System

Data obtained from Blue Card Services indicates that, since the inception of the blue card system in 2001 until 30 June 2014, there were 8,604 applications received from Indigenous persons located in communities with AMPs. In all, 6,731 PNBC and exemptions were issued to these applicants with 2,851 being issued where a criminal history was evident.

*“The challenges with these communities have always been the blue cards. People won’t appeal the decisions. We have repeat offenders from the communities, for violence. But the violence came from years and years ago so they continue to be penalised.” (Government)*

The impact of minor criminal histories on an individual’s eligibility to hold a PNBC together with the submissions process that affords applicants natural justice needs to be better understood in communities, particularly those in a position to advise community members.

## Discussion and recommendations

Our evidence indicates that some Indigenous residents of AMP communities in Queensland, and many representatives of the key agencies providing support and advice, including those with a mandate for children’s wellbeing, *perceive* that a conviction for a breach of AMP provisions, renders them ineligible to apply for and hold a PNBC. The implications of this are very serious for Indigenous children who have more frequent contact with child protection systems than non-Indigenous children (Australian Institute of Health and Welfare, 2015) and who are over-represented in child protection and out-of-home care (Australian Institute of Family Studies, 2015). The unnecessary removal of Indigenous children into foster care away from the community remains a major social challenge for Australia (Dudgeon et al., 2010; Libesman, 2004; Stanley et al., 2003).

There is an urgent need to develop and implement strategies to address the issues we have identified, to clarify the processes and requirements for both community members as well as the key stakeholders and service providers who hold the mandate and who have the obligation to advise clients reliably. Targeted education in these communities with these stakeholders must be developed urgently. Such strategies need careful consideration to ensure that all children remain safe and that the child protection system’s principles and functions are not undermined. Robust evaluation is also required to ensure that targeted education is reaching its intended beneficiaries and that it is taken up to correct the misconception.

According to the Australian Institute of Health and Welfare, 2014, the protection of children from physical and emotional harm is achieved through secure family and community environments, with the risk of harm increasing when children are exposed to substance misuse, racism, inadequate housing and social and economic disadvantage (Australian Institute of Family Studies, 2015; Australian Institute of Health and Welfare, 2014). AMPs were a Queensland Government initiative designed and implemented specifically for the discrete Indigenous communities, with the protection of children at the forefront of the reasoning underpinning their design. Our research raises questions, about the necessity for harsh penalties and consequences under the *Liquor Act* given the confusion in communities surrounding the ability to become carers, work in child related employment and/or obtain work at all.

Going forward, Government policies and programs that minimise these unanticipated consequences will be required to provide benefits for the affected communities and services. Policies and programs must give consideration to whether a criminal conviction should be recorded for a breach of an AMP, or whether it is more appropriate for convictions to be reserved for repeat/severe offenders.

Focused evaluation and research is needed in this important policy area to ensure Indigenous community members understand the reasons why a person may be refused a PNBC, as on its own, a breach of the *Liquor Act 1992* does not impact a person’s eligibility.
